# Bacterial communities in full-scale wastewater treatment systems

**DOI:** 10.1007/s11274-016-2012-9

**Published:** 2016-03-01

**Authors:** Agnieszka Cydzik-Kwiatkowska, Magdalena Zielińska

**Affiliations:** Department of Environmental Biotechnology, University of Warmia and Mazury in Olsztyn, Słoneczna 45G, 10-709 Olsztyn, Poland

**Keywords:** Activated sludge, Aerobic granules, Biofilm, Diversity, High-throughput sequencing, Microbial communities

## Abstract

Bacterial metabolism determines the effectiveness of biological treatment of wastewater. Therefore, it is important to define the relations between the species structure and the performance of full-scale installations. Although there is much laboratory data on microbial consortia, our understanding of dependencies between the microbial structure and operational parameters of full-scale wastewater treatment plants (WWTP) is limited. This mini-review presents the types of microbial consortia in WWTP. Information is given on extracellular polymeric substances production as factor that is key for formation of spatial structures of microorganisms. Additionally, we discuss data on microbial groups including nitrifiers, denitrifiers, Anammox bacteria, and phosphate- and glycogen-accumulating bacteria in full-scale aerobic systems that was obtained with the use of molecular techniques, including high-throughput sequencing, to shed light on dependencies between the microbial ecology of biomass and the overall efficiency and functional stability of wastewater treatment systems. Sludge bulking in WWTPs is addressed, as well as the microbial composition of consortia involved in antibiotic and micropollutant removal.

## Introduction

Operating parameters of wastewater treatment influence the formation of complex microbial structures and their species composition. The species structure of biomass determines metabolic pathways that may occur in the technological system and finally the quality of treated wastewater. To investigate the relationships between microorganisms responsible for pollutant removal from wastewater, emerging molecular biology techniques such as high-throughput sequencing are used. This study reviews the results obtained in full-scale wastewater treatment plants (WWTPs) because these results are the most valuable for process control, optimization and widening knowledge about the ecology of microorganisms involved in pollutant removal.

## Types of bacterial communities

In WWTPs, microorganisms are organized in species-rich structures that biodegrade a wider range of substrates than pure cultures. The formation of microbial aggregates is connected with production of extracellular polymeric substances (EPS). EPS are substances of biological origin that are created by cell lysis, secretion, shedding of material from cell surfaces and absorption of substances from the environment. Proteins and polysaccharides in the form of colloids are major constituents of EPS and determine biomass structure and properties. EPS production can be stimulated by the operating parameters of the treatment. Starvation in the long cycle length, a low COD/N ratio in the feed and a high nitrogen load force the use of organics for the production of EPS to maintain the structure of the biomass and to protect cells against the harmful effects of free ammonium and free nitrous acid (Cydzik-Kwiatkowska et al. 2014). EPS create a diffusion barrier that reduces the concentration of toxic compounds in the vicinity of cells. Microorganisms embedded in the EPS matrix complement each other’s functions. In this matrix, novel exopolysaccharide monomers such as uronic acids are detected which are not present in pure strain samples, which indicates the role of microbial interactions in WWTPs (Andersson et al. 2011).

Flocs of activated sludge are shaped by physico-chemical adhesion of cells and pollutants from wastewater, as well as by the formation of colonies of microorganisms. Bacteria constitute from 5 to 20 % of organics in flocs; the rest are EPS (Raszka et al. 2006). The size, density, shape and content of filamentous bacteria determine flocs stability and settling. Environmental stress may lead to floc fragmentation and increase the suspended solids concentration in the effluent (Henriques and Love 2007).

Biofilm is a multi-layer structure of microorganisms that are immobilized on solid supports via electrostatic interactions, covalent bonds, and hydrophobic interactions. Colonization is facilitated by fimbriae, cilia, cell wall components and EPS. Mass transport in the biofilm is predominantly due to diffusion, and the thickness of the biofilm is determined by the depth to which the substrate and oxygen can penetrate. The biofilm is crossed by channels and pores that facilitate genetic transfer between bacteria, e.g. by encouraging the spread of genes through horizontal transfer. Genetic changes in immobilized cells can increase their growth rate, metabolic activity and resistance to toxic compounds (Cohen 2001; Shuler and Kargi 2002). Activated sludge and biofilm differ in EPS composition within the same systems (Mahendran et al. 2012). In floc-derived EPS, proteins predominate whereas in biofilm-derived EPS, proteins and polysaccharides are present in similar proportions.

Aerobic granules are an example of microbial self-immobilization. Aerobic granular sludge technology allows the use of smaller reactors due to good settling ability of granules and a high concentration of microorganisms in their structure. Aerobic granulation involve initiation of physical contact between bacterial cells, formation of the granule structure due to microbial competition and changes in cellular metabolism and determination of granule morphology by hydrodynamic shear in the reactor. Aggregation and formation of granules is promoted by denitrification and the presence of slow-growing bacteria in the biomass (Liu et al. 2004; Wan and Sperandio 2009). The energy-rich substrates (glucose/fructose) stimulate the growth of filaments and formation of granules with a loose structure (Moy et al. 2002). The granule size is correlated with organic load; too high load causes disintegration of granules (Lopez et al. 2009). The increase in the granule diameter limits the diffusion of nutrients; starving cells derive energy from EPS, which impairs the mechanical strength of granules. The outer layer of granule is inhabited by aerobic microorganisms, while in deeper layers facultative and obligatory anaerobes develop, which enables removal of carbon and nutrients in one-stage system.

## Microbial composition in WWTPs

Biological wastewater treatment systems have been designed mostly from an engineering perspective, but in fact, many aspects of the ecology and dynamics of microbial communities should be taken into consideration. Integrating theoretical ecology in the design and operation of WWTPs is important since it allows better prediction of microbial community assembly and possible variations in community structure and function in response to environmental changes. Light microscopic observation and culture-based studies do not allow identification of the predominant populations in WWTPs due to the different susceptibility of bacteria to growth on microbiological media. Molecular approaches used to study bacterial diversity in WWTPs in a cultivation-independent manner demonstrated that most of the suggested model organisms are of minor relevance in situ and that other microorganisms, often not yet culturable, are responsible for most key processes in WWTPs (Wagner and Loy 2002). High-throughput sequencing targeting conserved regions in microbial genomes is now regarded as most reliable and cost-effective method of species composition analysis of environmental samples (Vanwonterghem et al. 2014).

The dynamics of bacterial communities in engineered ecosystems are influenced by deterministic and stochastic factors (Ofiţeru et al. 2010). Deterministic factors include competition and niche-specific variables while stochastic factors include microbial dispersal by random events of colonization/extinction or fluctuations in the influent composition e.g. nitrogen and organics loads or presence of toxic compounds. The two main measurements applicable to spatial-scale biodiversity are alpha-diversity and beta-diversity. Alpha-diversity expresses the diversity of a population within a system; a community will have a high alpha-diversity, when there is a high number of species with a similar number. The beta diversity investigates the variations in community composition between two ecosystems and measures the turnover of species between two sites in terms of gain or loss of species (Jost 2007). Comparisons of alpha-diversity are univariate (two samples can have the same richness but not share any common taxa), while beta-diversity measures dissimilarity between samples. Biological interactions are dominant drivers in determining the bacterial community assembly in WWTPs, whereas environmental conditions partially explain phylogenetic and quantitative variances and indirectly influence bacterial assembly (Ju and Zhang 2015). It was also observed that larger reactors have less dynamic but more efficient and diverse communities (Valentin-Vargas et al. 2012).

In WWTPs, quantitative changes between autotrophic and heterotrophic bacteria are affected by wastewater characteristics, the type and operation of technological system or geographic location (Cydzik-Kwiatkowska et al. 2012; Zhang et al. 2012; Ma et al. 2013). In municipal WWTPs, the phylum Proteobacteria (21–65 %) predominates, of which Betaproteobacteria is the most abundant class, largely responsible for organic and nutrient removal; subdominant phyla are Bacteroidetes, Acidobacteria, and Chloroflexi (Nielsen et al. 2010; Nguyen et al. 2011; Wan et al. 2011; Hu et al. 2012; Wang et al. 2012). A survey in 20 WWTPs found that the most numerous bacterial genera were *Tetrasphaera*, *Trichococcus*, *CandidatusMicrothrix*, *Rhodoferax*, *Rhodobacter*, *Hyphomicrobium*, p-55-a5 belonging to *Firmicutes* and P2CN44 and B45 belonging to the phyla Chloroflexi (McIllory et al. 2015). Of the Fungi, Ascomycota was the most abundant phylum, constituting 6.3–7.4 % of microorganisms in WWTPs, while the predominant phylum within Archea was Euryarcheota (1.5 % of microorganisms, Wang et al. 2014b).

Molecular studies have shown that the microbial structure of biomass depends on the type of technological system hence by selection of the treatment line we can favor species structure of biomass that supports process stability and efficiency. The species composition in anaerobic/oxic (AO) and anaerobic/anoxic/oxic (A^2^O) systems was more even than in membrane bioreactors (MBRs) or oxidation ditches (Hu et al. 2012). In MBRs, the phyla Bacteroidetes was quite homogenous and the order Sphingobacteriales predominated. The low diversity in MBRs can be caused by the long solid retention time (SRT), low food/microorganisms ratio or high availability of readily biodegradable organics (Hu et al. 2012; Wan et al. 2011). Although data on aerobic granulation in WWTPs are mainly focused on technological research (Pronk et al. 2015), Li et al. (2014) reported that *Flavobacterium* sp., *Aquabacterium* sp. and *Thauera* sp. occurred in granules and *Flavobacterium* sp. supported granule formation due to EPS production.

Molecular studies identified core microbial communities in efficient activated sludge WWTPs; lack of these communities may point out to reasons for malfunctioning of wastewater-treatment system. Zhang et al. (2012) observed that the species composition of biomass varied with geographic location, although microbial genera *Zoogloea*, *Dechloromonas*, *Prosthecobacter*, *Caldilinea* and *Tricoccus* existed in all WWTPs. Sphingobacteriales, Anaerolineales, Rhodocyclales, Burkholderiales, Rhizobiales, Xanthomonadales, Verrucomicrobiales, Clostridiales, Planctomycetales and Myxococcales were common in activated sludge from 14 WWTPs and accounted for over 95 % of all sequences (Wang et al. 2012). Part of the effect of geographic location on species composition is due to the fact that the temperatures of wastewater treatment vary in different locations. In an A^2^O system with a predenitrification tank, Saprospiraceae and Alphaproteobacteria were temperature-sensitive, whereas Betaproteobacteria, Actinobacteria and Chloroflexi constituted half of the microbial population regardless of season (Muszyński et al. 2015).

Investigations of microbial communities can indicate groups important for efficient degradation of recalcitrant compounds in industrial streams. In WWTPs treating pharmaceutical, petroleum refinery, pet food and coking wastewater, Proteobacteria predominated (Ibarbalz et al. 2013; Ma et al. 2015). Biomass taken from coking WWTPs was similar at each taxonomic level, with *Thiobacillus*, *Comamonas*, *Thauera, Azoarcus and Rhodoplanes* being the dominant genera. Their presence can be explained by their ability to biodegrade specific components of the industrial wastewater. Interestingly, *Zoogloea*, *Prostheobacter* and Acidobacteria of Gp6 subgroups, were only a small fraction of the biomass from coking WWTPs, even though they are regarded as core genera in municipal WWTPs (Ma et al. 2015).

Filamentous bacteria are usually present in WWTPs in a low number supporting the formation of microbial structures. The excessive growth of these bacteria causes sludge bulking. Filamentous bacteria are characterized by a low diversity and both geographic location and technological process are responsible for their species structure (Guo and Zhang 2012; Mielczarek et al. 2012). The species composition of filaments varies in bulking and non-bulking periods. During non-bulking period in conventional and inverted A^2^O processes, a low abundance of Types 0092 and 0041 filaments was detected, while during the bulking period, *Microthrix parvicella* predominated (Wang et al. 2014a). A significant abundance of *M. parvicella* during bulking can cause a shift from Proteobacteria to Actinobacteria. This causes an accumulation of nitrogen at maintained efficiency of phosphorus removal (*M. parvicella* can behave as PAO). In globally distributed WWTPs, the percentage of filaments varied from 1.86 to 8.99 % and main groups were *Nostocola limicola* I and II, *Mycobacterium fortuitum*, Type 1863 *Acinetobacter* and *Microthrix parvicella* (Guo and Zhang 2012). Other studies indicated *Microthrix* sp, *Haliscomenobacter hydrossis*-like bacteria and Types 0803 and 0092 belonging to phylum Chloroflexi as predominating filaments in municipal and domestic WWTPs (Mielczarek et al. 2012; Kowalska et al. 2015). Identification of filaments responsible for bulking enables to select optimal solution for their removal from a particular WWTPs.

Molecular methods enable to conclude about the fate of pathogens in wastewater treatment systems. Lu et al. (2015) have observed that *Arcobacter butzleri*, *Aeromonas hydrophila* and *Klebsiella pneumoniae* present in wastewater were efficiently eliminated during biological treatment. Analysis of the influent, activated sludge and the effluent of two WWTPs indicated that nine identified pathogens constituted about 0.06–3.20 % of total bacteria (Cai and Zhang 2013). In activated sludge and the effluent, similar pathogens, including *Mycobacterium tuberculosis*-like species, were identified and they were different from those in the influent. In the effluent from the WWTP treating salty wastewater, *Mycobacterium* sp. and *Vibrio* sp. were present; among these genera pathogenic microorganisms were numerous (Ye and Zhang 2013).

### Microorganisms in nutrients removal

Substrate affinity of nutrient-removing bacteria determines their species composition (Muszyński et al. 2015). Ammonia-oxidizing bacteria (AOB) diversity is higher in domestic WWTPs in comparison with municipal WWTPs and *Nitrosomonas* sp. are mainly responsible for nitrification (Limpiyakorn et al. 2006; Zhang et al. 2011b).

The temperature is regarded as one of the most important factors that affect AOB abundance and the balance between *Nitrosospira* sp. and *Nitrosomonas* sp. in WWTPs (Siripong and Rittman 2007; Cydzik-Kwiatkowska et al. 2012). A combination of low temperature and high SRT may favour *Nitrosospira* sp. (Siripong and Rittman 2007). The type of treatment system influences AOB sensitivity to temperature; AOB in conventional activated sludge reactors are more susceptible to seasonal variations than in MBR (Wan et al. 2011). Regarding nitrite-oxidizing bacteria (NOB), the growth of *Nitrospira* sp. in WWTPs is favored by low dissolved oxygen and short SRT, whereas the abundance of *Nitrobacter* sp. increases in winter season, in which dissolved oxygen is high (Huang et al. 2010). Lücker et al. (2015) have identified the novel NOB *CandidatusNitrotoga arctica* in activated sludge samples from 20 WWTPs. *Nitrotoga*-like bacteria either coexisted with *Nitrospira* sp. or were the only detectable NOB in abundance comparable to *Nitrospira* sp. abundances in other WWTPs. *Nitrotoga* sp. remained active at different nitrite concentrations and their presence was favored by low temperatures.

Autotrophic ammonia oxidation also occurs in the domain Archaea. The balance between ammonia-oxidizing archaea (AOA) and AOB in biomass depends on the influent ammonium concentration. In industrial WWTPs treating high-ammonium wastewater, the abundance of AOB was much higher than that of AOA, while in the municipal WWTPs receiving low ammonium influent, a significant abundance of AOA genes occurred (Limpiyakorn et al. 2011; Bai et al. 2012). The lower percentage of AOA in the industrial WWTPs indicates higher sensitivity of AOA to toxic compounds than AOB.

Successful nitrification despite the low abundance of autotrophic nitrifiers indicates ammonium removal in a heterotrophic process. The growth of heterotrophic nitrifiers from genera *Comamonas,Thauera, Paracoccus* and *Azoarcus* was observed in the activated sludge reactors treating ammonium-rich, high-organic tannery and coking wastewater (Wang et al. 2014c; Ma et al. 2015). *Pseudomonas* sp. and *Paracoccus* sp. conducted heterotrophic nitrification at high nitrogen load in laboratory-scale aerobic granules (Cydzik-Kwiatkowska 2015).

Denitrifiers belong to a broad variety of phylogenetic groups therefore they are difficult to investigate. In WWTPs, denitrifiers from *Thauera* sp. are usually detected (Jiang et al. 2008). Denitrifiers’ community in activated sludge is influenced by a type of technological system; the highest diversity of N_2_O-reducers was detected in the WWTPs with separated denitrification tanks (Jaranowska et al. 2013). Measurements of denitrifiers activity in biomass in the reactor cycle allow to conclude which operational parameters promote full denitrification and minimize nitrous oxide emission. Cydzik-Kwiatkowska and Wojnowska-Baryła (2015) observed that variable oxic conditions in the cycle of batch reactor with aerobic granules stimulated the activity of N_2_O-reducers and that the activity of nitrogen-converting bacteria was the highest with a 13-h hydraulic retention time. Different substrate requirements of nitrifiers and denitrifiers influence their spatial arrangement in the reactor. Relative abundance of nitrifers increased along the plug flowpath of rotating biological contactors, while the abundance of denitrifiers belonging to the genera *Rhodanobacter*, *Paracoccus*, *Thauera*, and *Azoarcus* markedly decreased (Peng et al. 2014).

To efficiently remove ammonium from highly-concentrated streams, Anammox process in side-stream and main-stream of WWTPs was developed (van der Star et al. 2007; Lackner et al. 2014). A shift from *Kuenenia stuttgartiensis* in the inoculum to *Brocadia anammoxidans* during the reactor operation suggested that niche differentiation determines species composition of Anammox systems (van der Star et al. 2007). The metagenomic survey of Anammox-enriched WWTPs with different operating conditions, system configurations, and influent characteristics revealed unique but complex community structure predominated by *Nitrosomonas* sp. and *Candidatus Kuenenia* (Park et al. 2014). AOB and Anammox bacteria are differently distributed in biomass. In one-stage nitritation-Anammox systems, flocs were mainly inhabited by AOB, while granules had AOB on outer surface and Anammox bacteria in an internal zone (Chu et al. 2015). *Nitrosomonas* sp. was the only AOB, while Anammox bacteria were *Candidatus Jettenia* (16.8 %) followed by *Candidatus Brocadia* (3.3 %). Despite strictly autotrophic wastewater composition, in Anammox reactors heterotrophic bacteria, e.g. *Chloroflexi* sp., were abundant that fed on soluble microbial products and EPS.

The enhanced biological phosphorus removal (EBPR) is conducted by polyphosphate accumulating organisms (PAOs). Their growth is stimulated by intermittent anaerobic/aerobic conditions. Some PAOs are able to remove phosphate using nitrite or nitrate as electron acceptors in denitrification (denitrifying polyphosphate accumulating organisms, DNPAOs) and use the same pool of organics to remove N and P (Carvalho et al. 2007). An abundant PAO in full-scale systems are *Accumulibacter* sp. that conduct anaerobic volatile fatty acids uptake and polyhydroxyacids (PHAs) storage, coupled with phosphate release and glycogen degradation (Oehmen et al. 2007). Other important contributors to phosphorus removal are *Tetrasphaera*-related PAOs that do not store PHA and take up amino acids instead of volatile fatty acids. *Tetrasphaera* sp. can comprise up to 30–35 % of the microbial community (Nielsen et al. 2010; Nguyen et al. 2011), whereas *Accumulibacter* sp. are usually less numerous (3–10 %) (Gu et al. 2008; He et al. 2008). The presence of both genera in biomass depends on high influent C/P ratio and high organic loading for *Accumulibacter* sp. and *Tetrasphaera* sp., respectively (Mielczarek et al. 2013). Another group of PAOs are *Dechloromonas*-related bacteria capable of acetate uptake, polyphosphate and PHA storage and nitrate/nitrite reduction; these bacteria were however found in EBPRs in low number (≤3 %) and diversity (Kong et al. 2007; Nielsen et al. 2010).

Most of heterotrophic bacteria is outcompeted by PAOs because PAOs can accumulate substrates and better survive starvation periods. The main competitor of PAOs are glycogen-accumulating organisms (GAOs) that do not accumulate phosphorus but compete with PAOs for volatile fatty acids (Gu et al. 2008). The abundant GAOs in full-scale EBPRs are *CandidatusCompetibacter phosphatis* (≤12 %) and *Defluviicoccus vanus* (9 %) (Saunders et al. 2003; Burow et al. 2007). In WWTPs with the abundance of *Accumulibacter* sp. and *Competibacter* sp. of about 4.8 %, the required level of phosphorus removal was not achieved (Zhang et al. 2011a). On the other hand, the investigations of biomass in 28 municipal EBPRs showed that PAOs and GAOs accounted for about 30 % of microbial community and the presence of GAOs (10–15 %) did not correlate with poor performance of EBPRs (Mielczarek et al. 2013).

### Microorganisms in micropollutant removal

Regarding metabolism of micropollutants in WWTPs, it was suggested that long SRTs enable efficient nitrification that improves micropollutants removal by co-metabolism (Clara et al. 2005). The potential of nitrifying immobilized biomass to remove polycyclic aromatic hydrocarbons (PAHs) and bisphenol A (BPA) from wastewater was proved at a short hydraulic retention time (Zielińska et al. 2012, 2014). BPA biodegradation is conducted by *Pseudomonas paucimobilis* (Ike et al. 1995), *Sphingomonasbisphenolicum* AO1 (Oshiman et al. 2007) or *Sphingomonas* sp. strain AO1 (Sasaki et al. 2005). Investigation of genes of *bisd* operon, coding enzymes responsible for initial BPA hydroxylation, allows identification of BPA-biodegrading microorganisms (Sasaki et al. 2005). BPA removal was associated with the activity of heterotrophic bacteria and exposure of biomass to BPA decreased the AOB number in the biofilm (Zielińska et al. 2014). Studies in WWTP showed BPA degradation by laccases produced by *Sinorhizobium meliloti* in raw and pretreated sewage sludge (Mohapatra et al. 2010).

Inefficient degradation of antibiotics results in the development of antibiotic-resistant bacteria (ARB) in the environment (Rizzo et al. 2013). Municipal WWTPs are the main sources of antibiotic-resistance genes (ARGs) therefore it is important to understand how the process design influences ARB abundance and distribution in wastewater treatment system. The dissemination of ARGs occurs mainly due to horizontal gene transfer (Munck et al. 2015). In the WWTPs, ARB abundance is the highest in the influent, followed by the effluent, anaerobic digestion sludge and activated sludge. Wastewater treatment can reduce over 99 % of ARB while the removal efficiency in sludge treatment is worse (Yang et al. 2014). Disinfection is a major tool to control the spread of ARB into the environment (Rizzo et al. 2013), however, e.g. chlorination can cause regrowth and reactivation of ARB in secondary effluents (Huang et al. 2011). The amount and kind of antibiotics in the influent decides about bacterial composition of the WWTP effluents. In WWTPs treating penicillin-containing wastewater, the phyla Proteobacteria and Firmicutes and bacteria from the classes Clostridia and Bacilli predominated in the effluents (Li et al. 2011). Novo et al. (2013) indicated that the occurrence of tetracyclines, penicillins, sulfonamides, quinolones and triclosan in the influent positively correlated with the abundance of Epsilonproteobacteria and negatively with Beta- and Gammaproteobacteria and Firmicutes. *Sulfuritalea*, *Armatimonas*, *Prosthecobacter*, *Hyphomicrobium*, *Azonexus*, *Longilinea*, *Paracoccus*, *Novosphingobium* and *Rhodobacter* were identified as potential tetracycline resistant bacteria. Treatment of high-tetracycline wastewater increased both the abundance and diversity of the *tet* genes but decreased the occurrence and diversity of non-tetracycline ARGs (Huang et al. 2014).

### Assessment of microbial activity in WWTPs

Next-generation sequencing has the potential to study the metabolic function of microbial consortia on the basis of gene expression. Comparison of metagenomic and metatranscriptomic datasets allow to assess the relative activity of microbial populations in WWTPs and conclude about genes that are the most active during pollutant removal. Study on activated sludge from municipal WWTP showed that Proteobacteria, Actinobacteria, Bacteroidetes, Firmicutes and Verrucomicrobia phyla predominated in both DNA and cDNA sets (Yu and Zhang 2012). Denitrification-related genes were most numerous in both DNA and cDNA datasets and the high cDNA/DNA ratios of nitrifying genes indicated strong ammonia oxidation activity. Analysis of sequences of nitrification genes showed that AOB mainly belonged to *Nitrosomonas* sp. and *Nitrosospira* sp., while AOA were absent in the biomass. Investigation of functional genes in biomass from WWTPs showed that 65–89 % of genes was shared between the samples indicating high similarity of microbial community functional structures in activated sludge (Wang et al. 2014b). Gene functional patterns correlated with wastewater temperature, dissolved oxygen and nitrogen and organic loads. Important genes such as *nosZ*, coding nitrous oxide reductase converting NO_2_ to N_2_, were highly diverse, which could promote stable denitrification.

## Summary

To improve wastewater treatment technology, an in depth understanding of microbial ecology and the web of intraspecies connections is needed. This goal seems within our reach, thanks to next-generation sequencing technologies that overcome the biases of both culture-dependent and PCR-based methods. Next-generation sequencing sheds light on microbial diversity and different functional genes in engineered environments. The data can be used to enhance bioaugmentation for the improvement of biodegradation of specific contaminants or wastewater treatment process modelling, monitoring and operation. Molecular data regarding the effect of operational parameters on microbial composition of biomass can support implementation in full-scale of sensitive technologies involving slow growing bacteria such as partial nitrification/Anammox process.

The biggest remaining challenge for next-generation sequencing is the development of bioinformatics tools that enable simple and reliable analysis of data on microbial communities in wastewater treatment systems. This problem has been addressed by many researchers and interesting projects, like the Microbial Database for Activated Sludge (MiDAS). MiDAS is a web platform that allows integration of data on the identity of abundant and process critical microorganisms in activated sludge wastewater treatment systems with information on their functional importance, morphology, diversity and distribution (McIllroy et al. 2015).
